# Impact of Health Related QoL and Mediterranean Diet on Liver Fibrosis in Patients with NAFLD

**DOI:** 10.3390/nu15133018

**Published:** 2023-07-02

**Authors:** Nuria Perez-Diaz-del-Campo, Gabriele Castelnuovo, Chiara Rosso, Aurora Nicolosi, Marta Guariglia, Eleonora Dileo, Angelo Armandi, Gian Paolo Caviglia, Elisabetta Bugianesi

**Affiliations:** 1 Department of Medical Sciences, University of Turin, 10126 Turin, Italy; nuria.perezdiazdelcampo@unito.it (N.P.-D.-d.-C.); gabriele.castelnuovo@unito.it (G.C.); chiara.rosso@unito.it (C.R.); aurora.nicolosi@unito.it (A.N.); marta.guariglia@unito.it (M.G.); eleonora.dileo@unito.it (E.D.); angelo.armandi@unito.it (A.A.); gianpaolo.caviglia@unito.it (G.P.C.); 2 Metabolic Liver Disease Research Program, I. Department of Medicine, University Medical Center of the Johannes Gutenberg-University, 55131 Mainz, Germany; 3 Gastroenterology Unit, Città della Salute e della Scienza—Molinette Hospital, 10126 Turin, Italy

**Keywords:** Health related QoL, mediterranean diet, NAFLD, liver fibrosis

## Abstract

Patients with non-alcoholic fatty liver disease (NAFLD) display impaired health-related quality of life (HRQoL) that is often linked to an unhealthy dietary pattern. The aim of this work was to investigate the impact of HRQoL and adherence to the Mediterranean diet on the risk of liver fibrosis (LF) in patients with NAFLD. LF was assessed in 244 patients through transient elastography (FibroScan^®^530. Echosens, Paris, France). Significant LF was defined according to liver stiffness measurements (LSM) values ≥ 7.1 kPa. The Mediterranean diet score and the Short Form-36 questionnaires were also completed. The median age was 54 (44–62) years and 57% of participants were male. A total of 42 (17.2%) participants had LSM ≥ 7.1 kPa and showed increased GGT (*p* = 0.001), glucose (*p* < 0.001), and triglycerides levels (*p* = 0.015) compared to those with LSM ≤7.0 kPa. Moreover, patients with significant LF had significantly lower scores related to Physical Functioning (*p* < 0.001) and Role Physical (*p* < 0.001). In the logistic regression analysis, lower role physical and lower adherence to the MedDiet (*p* = 0.001 and *p* = 0.009, respectively), after adjusting for age, diabetes, and obstructive sleep apnea, were associated with an increased risk of significant LF. Low adherence to MedDiet and low role physical may influence the risk of significant liver fibrosis in patients with NAFLD.

## 1. Introduction

Non-alcoholic fatty liver disease (NAFLD) is a prevalent liver condition characterized by the accumulation of excess fat in the liver, not caused by excessive alcohol consumption [1]. With its increasing prevalence globally, NAFLD has emerged as a significant public health concern [2]. Lifestyle factors play a crucial role in the development and progression of NAFLD, highlighting the importance of maintaining the high-quality lifestyle for both liver health and overall well-being [3].

Quality of life encompasses various dimensions including physical, mental, and social well-being [4]. NAFLD not only affects the liver but also has implications for an individual’s quality of life [5]. It can lead to complications such as liver inflammation (non-alcoholic steatohepatitis (NASH)), fibrosis, cirrhosis, and even liver cancer [6]. Additionally, NAFLD is closely associated with an increased risk of developing other metabolic disorders such as obesity, type 2 diabetes mellitus, and cardiovascular disease, further impacting quality of life [4,7].

Adopting a high-quality lifestyle can positively influence NAFLD outcomes and improve the overall quality of life [8,9]. Lifestyle modifications targeting weight loss, regular physical activity, and dietary changes have shown promising results in managing NAFLD [10]. In particular, adherence to a Mediterranean diet (MedDiet) has been shown to contribute to weight management, improved insulin sensitivity, and reduced inflammation, all factors that influence NAFLD progression and overall quality of life [11,12]. By adopting a high-quality lifestyle that includes regular physical activity, stress management, and adherence to a Mediterranean-style eating pattern, individuals with NAFLD can experience improvements in liver health, metabolic parameters, and the overall well-being [13].

Furthermore, addressing lifestyle factors beyond diet, such as stress reduction and adequate sleep, can have a positive impact on NAFLD outcomes and quality of life [14]. Chronic stress and poor sleep patterns have been linked to increased inflammation and metabolic dysfunction, which can exacerbate NAFLD [9]. Incorporating stress-management techniques and prioritizing sufficient sleep can contribute to better liver health and enhance overall quality of life [15].

Thus, maintaining a high-quality lifestyle is crucial for NAFLD prevention and management. The aim of this work was to investigate the possible association of health-related quality of life and adherence to the MedDiet with the risk of liver fibrosis in patients with NAFLD.

## 2. Materials and Methods

### 2.1. Study Design and Participants

From a cohort of consecutively enrolled patients with a diagnosis of NAFLD achieved by ultrasound (US) in the absence of other known causes of liver disease, the present study included patients enrolled between October 2019 and April 2023 at the outpatient clinic of the Unit of Gastroenterology, Città della Salute e della Scienza di Torino–Molinette Hospital. We included in the analysis patients who underwent vibration controlled transient elastography (VCTE) with assessment of controlled attenuation parameter (CAP) (FibroScan^®^530. Echosens, Paris, France), who were administered the Short Form-36 (SF-36) and the Mediterranean Diet Score (MDS) questionnaires. Alcohol-induced liver disease was excluded by selecting patients with a negative history of alcohol abuse, defined as a weekly ethanol consumption of <140 g for women and <210 g for men [16]. Subjects aged ≥ 18 years signed the informed consent for participation in the study.

### 2.2. Anthropometric and Biochemical Assessment

After an overnight fast, blood pressure, weight, height, and body mass index (BMI), calculated as the body weight divided by the squared height (kg/m^2^), were measured by qualified staff. Moreover, aspartate aminotransferase (AST), alanine aminotransferase (ALT) and gamma-glutamyl transferase (GGT), glucose, total cholesterol, and triglycerides concentrations were collected from patient’s medical records.

### 2.3. Assessment of Heath Related Quality of Life (HRQoL) and Dietary Intake

The generic SF-36 questionnaire assesses 8 Patient Reported Outcomes (PROs) domains (range 0–100): Physical Functioning (PF), Role Physical (RP), Bodily Pain (BP), General Health (GH), Vitality, Social Functioning, Role Emotional (RE), and Mental Health (MH). The remaining item (Health change) assesses changes in perceived health during the last year. Subscale scores range from 0 to 100, where higher scores represent better health status [17].

Furthermore, the MDS questionnaire was used to assess adherence to MedDiet. The questionnaire is based on a 14-point screening, ranging from 0 to 14, where a higher final score indicates better adherence to the Mediterranean diet [18].

### 2.4. Evaluation of Liver Status

VCTE and CAP were performed after overnight fasting to assess steatosis and possible liver stiffness measurement (LSM) on supine subjects with the right arm under the head and the right leg crossed with the left one. The examination probe (M or XL) was chosen according to the body condition between the 6th and 9th intercostal space. At least 10 snapshots of valid values were taken, of which the median was selected, and technically reliable measurements were considered on the basis of the interquartile range (IQR)/mean of less than 30%. LSM was reported as kPa while CAP values as dB/m; the degree of liver fibrosis was classified into two groups (LSM ≤ 7.0 kPa vs. LSM ≥ 7.1 kPa) according to established cut-offs [19].

### 2.5. Statistical Analysis

Continuous variables are expressed as means and standard deviation (SD) or as medians and interquartile ranges (IQR) depending on its distribution, while qualitative categorical variables were analyzed with the X2 test and reported as absolute (N) and relative frequencies (%). Distribution of variables was assessed through the Shapiro–Wilk test. Data normality and outliers were also checked using boxplots. Those variables following a normal distribution were analyzed using parametric statistical tests while for those variables with a non-normal distribution, non-parametric statistics were applied.

Descriptive statistics were used to compare baseline data of participants. For continuous variables, Student’s t-tests (for parametric) of independent samples and Mann–Whitney U tests (for non-parametric) were applied. Liver fibrosis was classified in LSM ≤ 7.0 vs. LSM ≥ 7.1 according to established cut-offs for significant fibrosis [19].

Then, univariate and multiple backward logistic regression models were set up to evaluate the association of significant liver fibrosis risk (dependent variables) with SF-36 components (independent variable). SF-36 components were divided according to the median value. Data were expressed in Odds Ratio (OR) and confidence interval.

Analyses were performed using STATA 12.0 software (Stata Corp College Station, TX, USA). All calculated *p*-values were two-tailed. Values of *p* < 0.05 were considered to be statistically significant in the analyses.

## 3. Results

### 3.1. Clinical, Biochemical and Hepatic Features of the Study Population

A total of 244 participants with US-diagnosed confirmed NAFLD were included in the study. Main body composition and biochemical features are reported in Table 1. Median age was 54 years (range 44–62) and most of the individuals were males (56.96%). Overall, the median BMI was 29.6 (range 26.8; 32.7) while most of the participants had a high school diploma or general equivalency diploma (GED) (58.2%). Furthermore, the main comorbidities were dyslipidemia (63.93%), followed by hypertension (49.59%) and diabetes (25%).

Concerning indirect assessment of liver damage, liver stiffness had a median value of 5.2 (range 4.3; 6.3), CAP 302 (range 263.5; 339.5), AST 29 (range 23; 39), ALT 39 (range 27; 31), and GGT 40 (range 26; 75). HRQoL levels were described as the outcome of the eight categories of the SF-36. The lowest median values were recorded in the domains of general health (points: 55 (range: 42.5; 70)) and health change (points: 50 (range: 37.5; 50)).

### 3.2. Characteristics of the Study Cohort according to Liver Stiffness Degree (Absent vs. Significant)

For the purpose of the analysis, participants were split into those with no liver fibrosis (LSM ≤ 7.0 kPa, *n* = 202) *versus* those with significant liver fibrosis (LSM ≥ 7.1 kPa, *n* = 42). Baseline anthropometric, biochemical and hepatic data as well as dietary intake characteristics of the study cohort according to hepatic steatosis groups are presented in Table 2. No sex differences were found between groups, while median age was significantly higher in the significant liver fibrosis group. Among the different educational levels, participants with LSM ≤ 7.0 kPa showed a higher percentage of university or higher education than those with LSM ≥ 7.1 kPa.

Also, patients with LSM ≥ 7.1 kPa had a higher rate of diabetes (*p* < 0.001), hypertension (*p* = 0.001) and obstructive sleep apnea (OSAS) (*p* = 0.001). As expected, participants with LSM ≥ 7.1 kPa displayed significantly increased CAP (*p* = 0.001), GGT (*p* = 0.001), glucose (*p* < 0.001), and triglycerides (*p* = 0.011), while lowers cholesterol levels were observed (*p* = 0.008). Regarding adherence to the MedDiet, no significant differences were found between groups.

**Figure 1:**
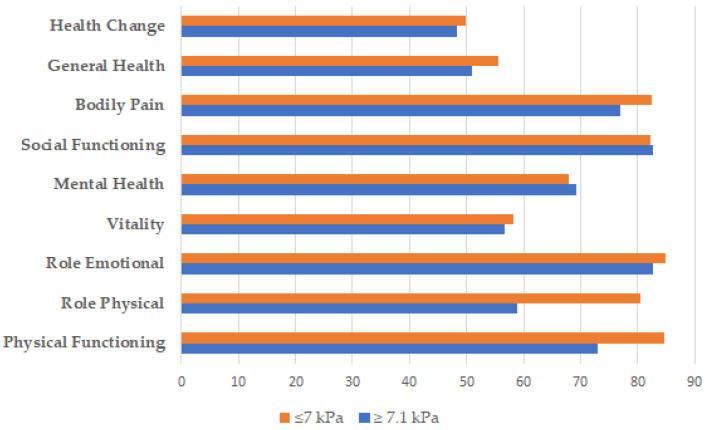
Mean SF-36 components (on a universal 0–100 scale) according to liver fibrosis degree.

In addition, LSM ≥ 7.1 kPa showed lower scores on almost all components of the SF-36 questionnaire. Particularly, significant differences were observed in Physical Functioning (84.7 vs. 72.9, *p* = 0.001) and Role Physical (80.4 vs. 58.9, *p* < 0.001) when comparing LSM ≤ 7.0 kPa vs. LSM ≥ 7.1 kPa, respectively (Figure 1).

### 3.3. Multiple Backward Regression Model for the Prediction of Significant Liver Fibrosis

In order to evaluate the risk of significant liver fibrosis a logistic regression model was constructed. To improve the prediction of the model, we built a multiple backward regression model, including high Physical functioning, together with the following variables: high Role Physical, Mediterranean diet score, sex, age, BMI, presence of diabetes, hypertension, and OSAS and education level. At multivariate analysis, we found that a high Role Limitations Physical and lower adherence to MedDiet were significantly associated to the risk of significant liver fibrosis, along with age, diabetes, and OSAS (Table 3).

## 4. Discussion

In this study, we demonstrated an association between quality of life, diet, and liver fibrosis in patients with NAFLD. Our data show that patients with significand liver fibrosis have a higher impairment of physical health-related outcomes in comparison with patients with LSM ≤ 7.0 kPa. The main drivers of impairment were Physical Functioning and Role Physical. Moreover, a higher Role Physical and a low adherence to MedDiet were able to predict the risk of significantly liver fibrosis. To our knowledge, this is the first investigation to proof the differential effect of quality of life and diet in patients with liver fibrosis diagnosed by VCTE.

In our cohort, patients with LSM ≥ 7.1 kPa, were older and a lower percentage had a high school graduate or GED compared to LSM ≤ 7.0 kPa. Patients with significant liver fibrosis also had a higher prevalence of comorbidities such as hypertension, dyslipidemia or diabetes, as well as higher biomarker values (i.e., AST, ALT, GGT, TG, glucose, and cholesterol). In this regard, patients with NAFLD older than 60 years have been found to have a higher prevalence of advanced fibrosis than younger patients [20]. In addition, type 2 diabetes has been associated with a more than two-fold increased risk of advanced fibrosis, cirrhosis-related complications and liver disease mortality as well as lipid abnormalities (such as low HDL cholesterol and high triglycerides levels) and hypertension [21]. Concerning the impact of Mediterranean diet, no significant differences were found among liver fibrosis groups in our cohort. In this regard, the Mediterranean diet is recommended by the EASL-EASD-EASO organizations for patients with NAFLD and has been shown to reduce liver fat even without weight gain [3,10]. Accordingly, a meta-analysis revealed that the MedDiet produced a significant decrease in serum triglyceride and total cholesterol levels, as well as a decrease in body weight and HOMA-IR compared to a control diet, in patients with NAFLD [22].

With regard to the SF-36 components, patients with NAFLD showed lower quality of life scores compared to other hepatic liver disease [5]. In our cohort, higher scores, meaning a best HRQoL were found in the absent liver fibrosis group. Specifically, significantly differences were observed in the Physical Functioning and Role Physical components between groups. In this regard, a cross-sectional study of 1338 patients with different liver diseases showed that NASH patients had significantly lower scores on Physical Functioning, Bodily Pain, General Health, Vitality, SF-36 Physical Summary and Fatigue [23].

Moreover, in our cohort, even if both the PF and RP domains of the SF-36 were significantly associated with the risk of LSM ≥ 7.1 kPa, when performing the multivariate logistic regression model, only limitations in usual role activities due to role physical remained significant, along with adherence to the MedDiet, age, diabetes, and OSAS. In this regard, although NAFLD is generally asymptomatic, fatigue is one of the common symptoms identified in these patients [24]. In fact, this symptom could be one of the explanations for the results observed in our patients. However, a recent study showed that only 75% of the NAFLD patients from the Global HGNA/NASH Registry had overt clinical fatigue recorded in their medical records, while the rest could only be elucidated through fatigue-specific PROs [25]. In line with our results, in a cohort of 509 biopsy-proven NAFLD patients with significant liver fibrosis referred to lower self-efficacy (*p* < 0.001), mental health (*p* < 0.001) and role-physical (*p* < 0.001), and more passive/avoidance coping (*p* = 0.002) [26]. In fact, poorer physical health and adherence to the MedDiet have been described as factors associated with liver fibrosis. Furthermore, although lifestyle changes are the main approach for NAFLD patients, few of them have currently access to personalized treatment in the public health system. Thus, there is a need for multidisciplinary teams including physicians, nurses, psychologists, nutritionists, and physical activity supervisors caring for patients with NAFLD to provide the best personalized approach [27]. Hence, efforts should be directed towards reducing the population’s exposure to an obesogenic environment, improving dietary, and lifestyle habits of the general population and facilitating healthy choices.

The main limitation of our study stems from its cross-sectional design, which does not allow conclusions to be drawn about the causal nature of the reported associations. Therefore, the present findings should be externally validated in a different cohort. Furthermore, the data collected in the questionnaires were self-reported, so there is a possibility that not all responses are completely accurate and, therefore, the results should be interpreted with caution. Lastly, we lack physical activity assessment in these patients as well as a NAFLD biopsy-proven group, so further studies should be carried out. Nevertheless, the consideration of both dietary and quality of life aspects of this work, as well as the population assessed by VCTE (FibroScan^®^530) can be considered the main strength of this study.

## 5. Conclusions

The current global epidemic of NAFLD makes this disease an important priority for healthcare and research. Our study confirmed the importance of both HRQoL such as role physical and dietary assessment in the management of patients with significant liver fibrosis.

Further studies are needed to evaluate the possible mechanisms by which dietary and lifestyle changes may influence the prevention, diagnosis, and progression of NAFLD.

## Figures and Tables

**Table 1:** General characteristics of participants.

*N*	244
Sex (Male/Female)	139/105
Age (years)	54 (44; 62)
BMI (kg/m^2^)	29.6 (26.8; 32.7)
Educational attainment, *n* (%)	
Less than high school graduate	57 (23.36)
High school graduate or GED	142 (58.2)
Some college or above	45 (18.44)
Main comorbidities	
Diabetes, *n* (%)	61 (25.0)
Arterial hypertension, *n* (%)	121 (49.59)
Dyslipidemia, *n* (%)	156 (63.93)
Depression, *n* (%)	16 (6.56)
Obstructive sleep apnea, *n* (%)	14 (5.74)
Liver and biomarkers assessment	
Liver stifness (kPa)	5.2 (4.3; 6.3)
CAP (dB/m)	302 (263.5; 339.5)
AST (U/L)	29 (23; 39)
ALT (U/L)	39 (27; 31)
GGT (U/L)	40 (26; 75)
Glucose (mg/dL)	97 (87; 112)
Triglycerides (mg/dL)	126 (96; 172)
Cholesterol (mg/dL)	198 (177; 226)
HRQoL assessment	
Physical functioning, points (IQR)	90 (75; 95)
Role Physical, points (IQR)	100 (50; 100)
Role Emotional, points (IQR)	100 (33.3; 100)
Vitality, points (IQR)	60 (47.5; 72.5)
Mental Health, points (IQR)	68 (56; 84)
Social functioning, points (IQR)	87.5 (62.5; 100)
Bodily Pain, points (IQR)	80 (57.5; 100)
General health, points (IQR)	55 (42.5; 70)
Health change, points (IQR)	50 (37.5; 50)

ALT, Alanine Aminotransferase; AST, Aspartate Aminotransferase; CAP, Controlled Attenuation Parameter; GED, General Equivalency Diploma; GGT, Gamma-Glutamyl Transferase; HRQoL, Health Related Quality of Life. Data are shown as n (percent) or median (IQR).

**Table 2:** Characteristics of participants according to the liver fibrosis degree.

	LSM ≤ 7 kPa	LSM ≥ 7.1 kPa	*p*-Value
*N*	202	42	
Sex, *n* (%)			
Male	115 (56.93)	24 (57.14)	0.980
Female	87 (43.07)	18 (42.86)	
Age (y)	51.5 (42;60)	62 (55; 66)	<0.001
Educational attainment			
Less than high school graduate, *n* = 57	44 (21.78)	13 (30.95)	0.178
High school graduate or GED, *n* = 142	117 (57.92)	25 (59.52)	
Some college or above, *n* = 45	41 (20.30)	4 (9.52)	
Main comorbidities			
Diabetes, *n* (%)	39 (19.31)	22 (52.38)	<0.001
Hypertension, *n* (%)	90 (44.55)	31 (73.81)	0.001
Dyslipidemia, *n* (%)	126 (62.38)	30 (71.43)	0.266
Depression, *n* (%)	12 (5.94)	4 (9.52)	0.393
Obstructive sleep apnea, *n* (%)	7 (3.47)	7 (16.67)	0.001
Body Mass Index (kg/m^2^)	29.4 (26.7; 32.2)	30.5 (26.3; 34.1)	0.185
CAP (dB/m)	297.5 (262; 332)	326.5 (292; 356)	0.001
AST (U/L)	29 (23; 38)	33.5 (25; 43.5)	0.094
ALT (U/L)	37 (25; 60)	45 (29; 61)	0.295
GGT (U(L)	37 (25; 71)	54.5 (38; 98)	0.001
Glucose (mg/dL)	95 (87; 107)	107 (95; 128)	<0.001
Triglycerides (mg/dL)	121 (92.5; 168)	154 (112; 192)	0.015
Cholesterol (mg/dL)	203 (179; 228)	185.5 (154; 203)	0.008
Adherence to MedDiet	7 (6; 9)	7 (5; 8)	0.073

ALT, Alanine Aminotransferase; AST, Aspartate Aminotransferase; CAP, Controlled Attenuation Parameter; GED, General Equivalency Diploma; GGT, Gamma-Glutamyl Transferase. Data are shown as N (percent) or median (IQR).

**Table 3:** Univariate and multivariate analysis on the effect of Mediterranean diet adherence, and SF-36 components for risk of significant liver fibrosis (LSM ≥ 7.1).

	Univariate Analysis		Multivariate Analysis	
	OR (95% CI)	*p*-Value	OR (95% CI)	*p*-Value
High Physical functioning	0.97 (0.95; 0.98)	0.001	-	-
High Role Physical	0.98 (0.97; 0.99)	0.001	0.27 (0.1; 0.6)	0.001
Mediterranean diet score	0.86 (0.7; 1.0)	0.066	0.77 (0.6; 0.9)	0.009
Female	0.99 (0.5; 1.9)	0.980	-	-
Age	1.0 (1.0; 1.1)	<0.001	1.07 (1.0; 1.1)	0.004
Body Mass Index	1.0 (1.0; 1.1)	0.033	-	-
Diabetes	4.5 (2.2; 9.2)	<0.001	3.83 (1.6; 8.6)	0.001
Hypertension	3.5 (1.6; 7.3)	0.001	-	-
Obstructive sleep apnea	5.5 (1.8; 16.8)	0.002	5.9 (1.5; 22.4)	0.008
Educational attainment				
Less than high school graduate, *n* = 57	*Reference*	-	-	-
High school graduate or GED, *n* = 142	0.7 (0.3; 1.5)	0.400	-	-
Some college or above, *n* = 45	0.3 (0.09; 1.0)	0.070	-	-

GED, General Equivalency Diploma.

## Data Availability

All data are publicly available.
